# Interfacial Stability of Acid–Crude Oil Emulsions in Matrix Acidizing of Carbonate Reservoirs

**DOI:** 10.1021/acsomega.5c08525

**Published:** 2025-10-31

**Authors:** Elisa Alves Mayrinck Macedo, Normann Paulo Dantas da Silva, Maria Carolina Neves Silva, Dennys Correia da Silva, Mateus Palharini Schwalbert, Alcides de Oliveira Wanderley Neto, Marcos Allyson Felipe Rodrigues

**Affiliations:** a Department of Chemical Engineering, 28123 Federal University of Rio Grande do Norte, Natal, Rio Grande do Norte 59072-970, Brazil; b Department of Petroleum Engineering, 28123 Federal University of Rio Grande do Norte, Natal, Rio Grande do Norte 59072-970, Brazil; c Postgraduate in Chemical Engineering, 28123 Federal University of Rio Grande do Norte, Natal, Rio Grande do Norte 59072-970, Brazil; d Petrobras/CENPES, Av. Horácio Macedo, 950 - C. Universitária - Ilha do Fundão, Rio de Janeiro, RJ, CEP: 21941-915, Brazil

## Abstract

Matrix acidizing is a fundamental technique for enhancing oil recovery in carbonate reservoirs by injecting acidic solutions, typically 15% (w/w) HCl, to promote wormhole formation and bypass damaged zones. However, acid–crude oil interactions frequently result in stable emulsions, leading to formation damage, reduced hydrocarbon mobility, and excessive acid consumption. This study presents a systematic investigation of the Emulsion Stability Index (ESI) in acid–oil systems under carefully controlled laboratory conditions designed to simulate potential field scenarios, assessing the effects of temperature (30–80 °C), acid-to-oil volumetric ratio (0.2–0.8), and FeCl_3_ concentration (0–3000 ppm). The experiments were conducted in triplicate to ensure reproducibility with no significant differences observed among replicates. The acid-to-oil ratio was identified as the most influential factor in mitigating emulsion formation. Lower ratios (e.g., 0.2) significantly reduced ESI values, particularly when combined with elevated temperatures and ferric ion concentrations. In addition, commercial additives—a demulsifier and a corrosion inhibitor, both characterized by high hydrophilic–lipophilic balance (HLB) and applied at concentrations of 1, 3, and 5 vol %—were evaluated for their performance. The main findings indicate that lower acid-to-oil ratios combined with elevated temperatures, FeCl_3_, and the presence of 3–5 vol % of these additives significantly reduce emulsion stability, enabling safer and more effective acidizing operations in carbonate reservoirs.

## Graphical abstract

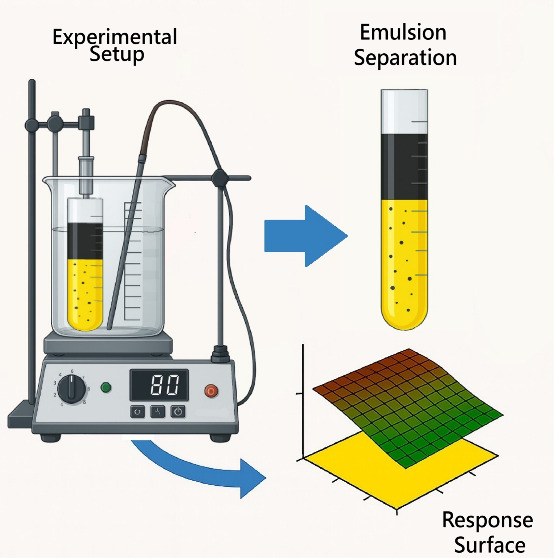

## 1 Introduction

Throughout the lifetime of an oil well, the production rate may experience declines due to various factors. Stimulation techniques are essential for enhancing oil flow and increasing production and operational profitability.^1–3^ However, beyond general formation damage, the interfacial behavior between crude oil and injected acid plays a decisive role in matrix acidizing performance.

During acidizing, hydrochloric acid (HCl) is injected to dissolve carbonate rock and create wormholes, enhancing permeability.^4–6^ Yet, when crude oils containing asphaltenes contact acidic solutions, complex acid–oil emulsions may form.^7^ These emulsions, stabilized by indigenous surfactants such as asphaltenes and resins, are remarkably persistent because asphaltenes accumulate at the oil–acid interface, creating rigid interfacial films that resist coalescence.^8–10^ This stability increases flow resistance, delays acid–rock contact, and can cause additional formation damage by blocking pores and trapping acid within the oil phase.^11^

The interfacial stability of these emulsions is governed by multiple operational and physicochemical factors.^12^ Temperature can influence droplet coalescence by altering the molecular mobility and interfacial tension. Acid-to-oil ratio affects emulsion type and inversion points, dictating whether water-in-oil (W/O) or oil-in-water (O/W) systems dominate. Ionic strength and the presence of multivalent ions such as Fe^3^^+^ modify electrostatic interactions at droplet surfaces, potentially destabilizing emulsions under certain conditions.^13–16^ Furthermore, corrosion inhibitors and demulsifiers, essential for protecting equipment and breaking persistent emulsions, directly impact interfacial stability. Asphaltene control also plays a pivotal role, as precipitation or adsorption phenomena exacerbate emulsion formation.^17,18^

Recent studies, such as those by Ala Al-Dogail et al.,^19^ investigated emulsified acid systems stabilized with organoclays (OCs) and demonstrated that modifying stabilizers improves thermal stability and reduces viscosity under high-shear conditions. Complementary work on carbon nanodots as drag-reducing agents further emphasized the importance of interfacial control in acidizing operations.^20^ While these approaches focus on engineered additives, the natural stabilization by indigenous surfactants in crude oils remains less explored.

In this context, the present work systematically evaluates the interfacial stability of acid–crude oil emulsions under controlled laboratory conditions. By examining key operational parameters—temperature, FeCl_3_ concentration, ionic strength, and acid-to-oil ratio—this study offers mechanistic insights directly relevant to field-scale operations. Rather than relying on novel stabilizers, our findings highlight practical adjustments that can minimize emulsion formation, reduce acid consumption, and improve the efficiency and sustainability of carbonate reservoir stimulation.

## 2 Materials and Methods

### 2.1 Preparation of the Acid Solution

Hydrochloric acid (37%, Synth) was diluted to a concentration of 15% (w/w) by adding distilled water. The final concentration of the HCl solution was validated through titration using a secondary standard (NaOH), which had been previously standardized with a primary standard (potassium biphthalate 99%, Synth).

### 2.2 Emulsification Evaluation System

The experimental setup is illustrated in Figure 1A (schematic) and Figure 1B (photograph). For the assembly of the experimental system, a 2 L beaker was filled with water and equipped with a magnetic stirrer. The beaker was then placed on a magnetic stirrer with a heater (Med Steel, XMTE-205). A stand with a rod and clamps was positioned next to the beaker and stirrer to support the graduated cylinder containing the mixture to be studied within the thermostatic bath as well as the thermometer.

**Figure 1:**
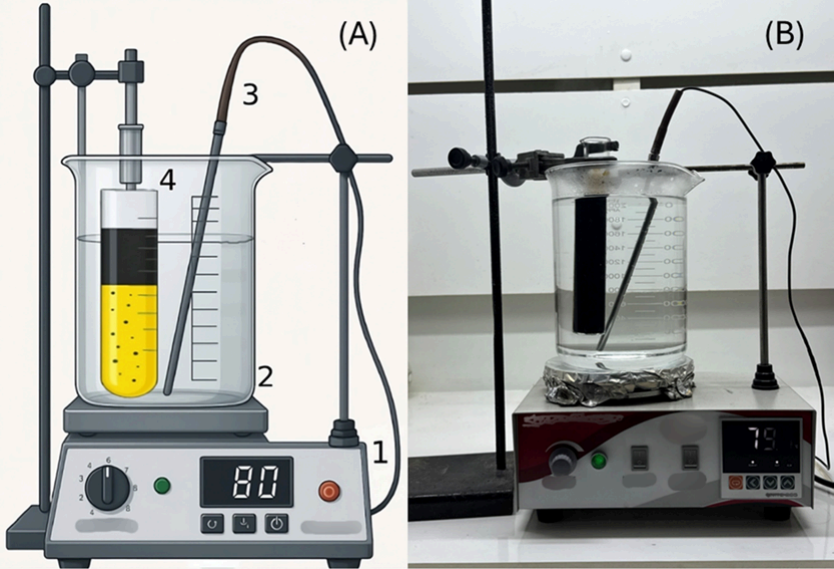
Experimental setup for evaluating the emulsification between crude oil and 15% (w/w) hydrochloric acid. (A) Schematic representation of the system, with numbered components: (1) magnetic stirrer with heating function; (2) thermostatic bath beaker; (3) thermometer; and (4) graduated cylinder for collecting the emulsified oil–acid mixture. (B) Photograph of the experimental apparatus.

The preparation of the emulsion began with the pipetting of a specific amount of 15% HCl solution into a 50 mL graduated cylinder, with the volume determined by the experimental design (to be introduced in Section 2.4). The HCl solution either contained or did not contain ferric chloride (FeCl_3_) (97%, Êxodo Cientfica), depending on the test condition. The presence of FeCl_3_ was investigated because ferric ions can interact with asphaltene molecules and acidic species via electrostatic and coordination interactions, influencing the emulsion stability. In addition, FeCl_3_ is relevant, as it can originate from the corrosion of metallic well tubing by hydrochloric acid during matrix acidizing, thereby representing a realistic operational scenario. Subsequently, a volume of crude oil was added, also determined according to the experimental design and carefully layered over the acid phase. The crude oil used in this study had been previously characterized by our research group (20.7° API, density of 0.929 g/cm^3^, and BSW of 0.3%).^21^

The mixture was then transferred to a Hamilton Beach mechanical stirrer (model HMD200) and stirred at a speed of 16,000 rpm for 5 min to form the oil–acid emulsion. The prepared emulsion was then carefully poured into a 50 mL graduated cylinder, avoiding air bubbles, and placed vertically into the thermostatic bath maintained by the 2 L beaker with water. The heating rate was not actively controlled, but the temperature was monitored continuously until the desired target was reached. Subsequently, each test was monitored for 60 min, starting from the moment the system attained the predefined temperature.

### 2.3 Emulsion Stability Index (ESI)

To assess the stability of the emulsions, the Emulsion Stability Index (ESI) was used, calculated as the percentage of the remaining acid phase volume in the emulsion relative to the initial volume of acid, as shown in eq 1.^22,23^ This index was obtained after 60 min of sedimentation at a fixed temperature, once the system had reached thermal equilibrium. The ESI measures the amount of acid retained in the emulsion after the test; a higher ESI indicates a greater amount of emulsified acid, while a lower ESI suggests a smaller amount of emulsified acid in the oil.

$$\mathrm{ESI}\left(\%\right)=\left(1-\frac{\mathrm{Separated}\;\mathrm{acid}\;\mathrm{volume}\;\left(\mathrm{ml}\right)}{\mathrm{Initial}\;\mathrm{acid}\;\mathrm{volume}\;\left(\mathrm{ml}\right)}\right)\times 100$$ 1

### 2.4 Experimental Design

To investigate the Emulsion Stability Index (ESI), the following variables were selected: ferric chloride III concentration (CFeCl_3_, ppm), temperature (*T*, °C), and oil/15% HCl solution ratio (*R*_oil_/HCl, w/w). To evaluate the effects of these variables and optimize the process, a full-factorial experimental design (2^3^) with three central point replications was implemented.^24^ The response variable was the Emulsion Stability Index (ESI). The levels of each variable and corresponding coded values are presented in Table 1.

**Table 1:** Levels of the Parameters Influencing the Studied System

	level		
variable	–1	0	1
C_FeCl_3__(ppm)	0	1500	3000
*T* (°C)	30	55	80
*R*_oil/HCl_	0.2	0.35	0.5

The variables were selected based on a thorough review of the literature, with values adjusted according to the conducted experiments. The temperature was defined based on the studies by Hedayati et al.^25^ and Yang et al.,^26^ which investigated temperatures of 30, 55, and 80 °C. The concentration of ferric chloride (FeCl_3_) was established according to the research by Abbasi et al.,^22^ considering operationally relevant levels in matrix acidizing ranging from 0 to 3000 ppm. This range allows for the evaluation of the effect of Fe^3^^+^ ions on emulsion formation and stability while representing realistic field conditions. Ferric chloride was chosen due to its role as a product of the corrosion of metal tubing in the well by hydrochloric acid. FeCl_3_ dissociates into Fe^3^^+^ and Cl^–^, and iron ions interact with asphaltene molecules, forming an emulsion with hydrochloric acid.^27,28^

Additionally, the oil/acid ratio was adapted from the studies by Hedayati et al.,^25^ which used ratios of 0.2, 0.5, and 0.8. During the experiments, it was observed that a ratio of 0.8 resulted in complete emulsification of the acid in the oil. This condition prevented measurable separation of phases and, consequently, the calculation of the ESI. Therefore, for the quantitative purposes of this study, the ratios were adjusted to 0.2, 0.35, and 0.5, corresponding to oil/HCl ratios of 1:5, 1:2.86, and 1:2, respectively, as higher values led to complete emulsification, preventing a detailed study of ESI variation, as seen in Figure 2.

**Figure 2:**
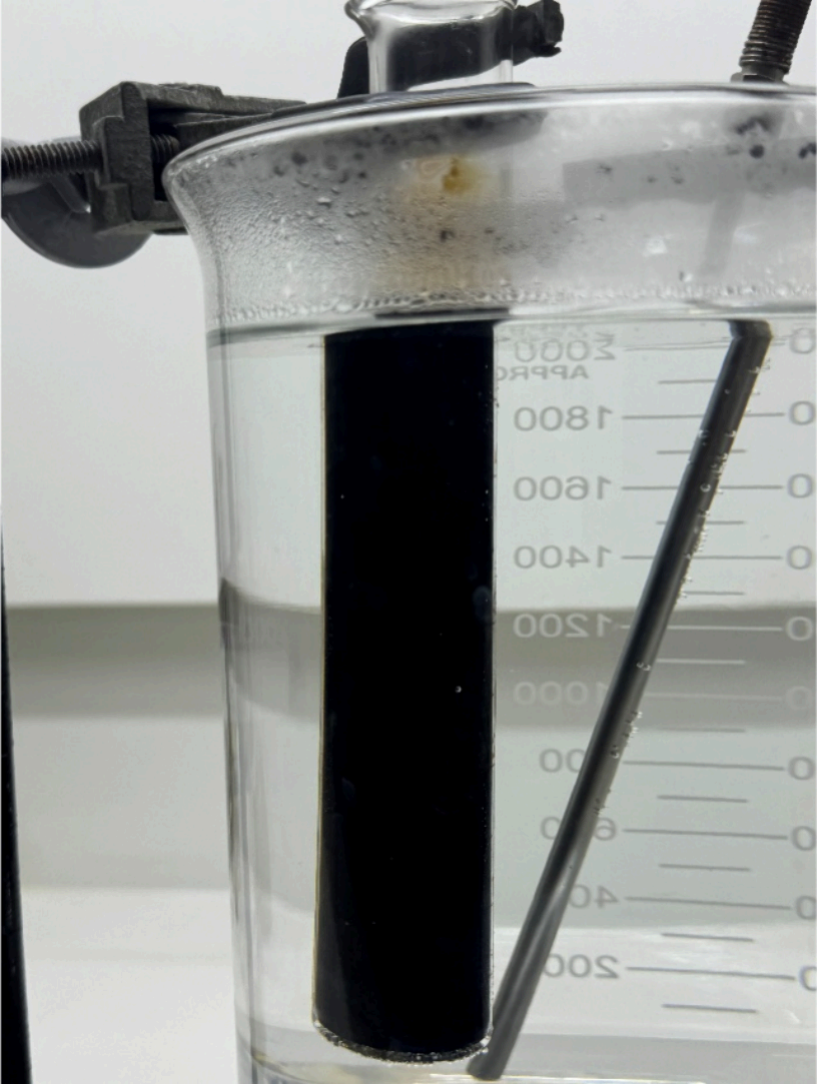
Acid–oil system exhibiting complete emulsification at an oil/HCl ratio of 0.8 (v/v), where no phase separation was observed.

In total, 11 experiments were conducted, varying between the minimum and maximum values defined in the experimental design. These experiments were essential in determining the optimal point for the lowest Emulsion Stability Index (ESI), indicating the smallest amount of emulsified acid in the oil. Each experiment was conducted in triplicate to ensure reproducibility. The experimental results were used to generate empirical response surfaces, which were modeled and analyzed by using STATISTICA 7.0 software.

### 2.5 Optimization Study

Based on the experimental design, all Emulsion Stability Index (ESI) values from the tests were analyzed to identify the condition that resulted in the minimal formation of the emulsified acid. This condition was defined as the optimal operating point for minimizing emulsion formation under the tested parameters. The optimal condition was subsequently adopted as a reference baseline for further experiments evaluating the effects of commercial additives widely applied in oilfield-acidizing operations, namely, a corrosion inhibitor and a demulsifier. The corrosion inhibitor corresponds to a nitrogen-based surfactant blend with amphiphilic character, typically classified within the cationic surfactant family, which is commonly employed to mitigate steel surface corrosion in acidic media. The demulsifier, in turn, is a nonionic surfactant formulation with a relatively high hydrophilic–lipophilic balance (HLB), designed to promote destabilization of acid–oil emulsions through interfacial tension reduction and coalescence of dispersed droplets. It is important to note that both additives were provided by a commercial supplier under confidentiality agreements, and therefore, specific molecular structures and precise physicochemical parameters could not be disclosed. Instead, only these general characteristics, as authorized by the manufacturer, are reported herein.

#### 2.5.1 Demulsifier Concentration

The effect of the commercial demulsifier on emulsion stability was evaluated through triplicate tests using concentrations of 1, 2, 3, 4, and 5 vol %. The previously determined optimal conditions served as a reference for these experiments. The demulsifier, characterized by a high hydrophilic–lipophilic balance (HLB > 12), was added to hydrochloric acid in a 100 mL volumetric flask, and the volume was completed with acid up to the mark. Due to confidentiality restrictions, the specific formulation and molecular structure of this additive cannot be disclosed. Subsequent procedures followed the methodology described in Section 2.2. During the ESI measurements, particular attention was given to the presence of dispersed oil droplets in the acid phase. These were visually monitored and excluded from the separated acid volume to avoid an overestimation of stability.

#### 2.5.2 Corrosion Inhibitor Concentration

The commercial corrosion inhibitor was studied using the same procedure as for the demulsifier, with triplicate tests using concentrations of 1, 2, 3, 4, and 5 vol %. The previously established optimal point was used as a reference for these experiments. This additive, known to possess surface-active properties and high HLB values, was mixed with hydrochloric acid in a 100 mL volumetric flask, and the volume was completed to the mark with acid. As with the demulsifier, the exact chemical composition and HLB value of the corrosion inhibitor are proprietary and are not authorized for publication by the supplier. Due to the low solubility of the inhibitor in inorganic acids, ultrasonic equipment was employed to homogenize the solution. Subsequent steps followed the procedure described in Section 2.2. As with the demulsifier tests, care was taken to visually inspect the acid phase after phase separation and exclude oil droplets from the separated acid volume measurement.

#### 2.5.3 Effect of Salinity

The effect of salinity was evaluated through tests with four salts: magnesium chloride (MgCl_2_), calcium chloride (CaCl_2_), sodium chloride (NaCl), and potassium chloride (KCl). These salts were selected due to their presence in the water, a common fluid in rock formations.^29^ Similarly to ferric chloride, these salts fully dissociate in aqueous media and influence the emulsification behavior of acid–oil systems through their ionic strength and specific ion effects. Tests were conducted by varying the salt molarity at 0.05 0.1, 0.5, and 1 M. Each experiment was performed in triplicate, with the previously established optimal condition serving as the baseline reference.

For the tests, the required amount of each salt was weighed and added to a 100 mL beaker. The acid was then pipetted into a beaker containing the salt, and the mixture was homogenized with a glass rod until complete dissolution of the salt. Subsequent steps followed the methodology described in Section 2.2. It is noteworthy that the effect of increasing salt concentration on the Emulsion Stability Index (ESI) exhibited a plateau behavior, where initial increases in ionic strength reduced electrostatic repulsion by screening the electrical double layer but further increases did not proportionally affect emulsion stability. This behavior was particularly observed for NaCl and KCl solutions at concentrations above 0.1 M, and it warrants further investigation to elucidate the underlying physicochemical mechanisms.

## 3 Results and Discussion

### 3.1 Analysis of the Experimental Design and Variable Interactions

Table 2 presents data related to the stability of the emulsions, taking into account the influence of ferric chloride (FeCl_3_) concentration, temperature, and oil/HCl ratio. Test 4, conducted with 3000 ppm ferric chloride at 80 °C and an oil/acid ratio of 0.2 (1:5), achieved the lowest Emulsion Stability Index (ESI), indicating the smallest amount of emulsified acid in the oil. The analysis of the variables at this optimal point revealed several relevant aspects that elucidate the observed behavior.

**Table 2:** Test Conditions and Experimental Design Results for ESI (%)

assay	C_FeCl3_ (ppm)	*T* (°C)	*R*_oil/hcl_	ESI (%)
1	0	30	1:5	28 ± 0.01
2	3000	30	1:5	28 ± 0.01
3	0	80	1:5	25.6 ± 0.00
4	3000	80	1:5	20.8 ± 0.01
5	0	30	1:2	64 ± 0.00
6	3000	30	1:2	58 ± 0.00
7	0	80	1:2	61 ± 0.01
8	3000	80	1:2	55 ± 0.01
9	1500	55	7:20	40.6 ± 0.00
10	1500	55	7:20	37.9 ± 0.00
11	1500	55	7:20	43.3 ± 0.00

Upon contact with the aqueous medium, oil formed an emulsion, particularly when it contained high concentrations of asphaltenes and resins. These fractions of petroleum contained molecules with oxygen, sulfur, and nitrogen atoms—heteroatoms with free electron pairs capable of attracting positive charges.^30,31^ When the aqueous medium contained cations, the asphaltenes and resins attracted these charges, imparting localized polarity to these molecules and behaving as natural surfactants with a low hydrophilic–lipophilic balance (HLB).^32^ These surfactants exhibited a greater affinity for oil than for water. In a mixture with a higher proportion of oil and a lower amount of acid, an environment conducive to the formation of a water-in-oil (W/O) emulsion was created, as the H^+^ ions from the acid interacted with the asphaltenes, forming interfacial films that stabilized inverse micelles.^14^ Furthermore, the acid used in stimulation could induce corrosion of metallic surfaces if the corrosion inhibitor was not fully effective, releasing Fe^3^^+^ ions, which enhanced the formation of native surfactants and, consequently, the stability of the emulsion.^33^

Conversely, when the mixture had a higher proportion of acid and a lower amount of oil, a scenario favorable to the formation of an oil-in-water (O/W) emulsion occurred. However, due to the low HLB of the formed surfactant, which favored the formation of inverse emulsions, the amount of emulsified system was determined by the amount of acidic solution present in the inverse micelle (water-in-oil), as the surfactant emulsified under these conditions.^34,35^ With an excess of acidic solution in the medium, the chemical system was separated into two phases because the interfacial films formed by asphaltenes and resins reached their saturation capacity. Once the micelles could not incorporate additional aqueous phase, the excess acid segregated as a free continuous phase, while the stabilized fraction remained dispersed as droplets in oil. This behavior was consistent with previous observations by Ganeeva et al.,^7^ who reported similar phase separation in emulsified acid systems once their stabilizing capacity was exceeded. The addition of ferric chloride to the medium induced the release of Fe^3^^+^ ions, which were attracted to the free electrons in the resin and asphaltene structures. This increased the polarity of the indigenous surfactant, enhancing its efficiency but simultaneously limiting the emulsification of larger amounts of acidic solution as the micelles reached their saturation capacity. Thus, the presence of Fe^3^^+^ ions, which might be expected to increase emulsification of additional aqueous phases, ultimately contributed to emulsion destabilization. These ions interacted with hydrocarbons outside the micelle, forming aggregates that coalesced into oil droplets, which separated easily from the acidic aqueous medium.^22,27^

Another notable point was that increasing the temperature reduced the level of emulsion formation. The increase in the thermal energy in the system caused enhanced molecular mobility of the acid and oil molecules, weakening their interactions. Experimental data indicated that a temperature of 80 °C significantly disrupted emulsions, as evidenced by the decrease in ESI.^36,37^ This phenomenon could be explained by the theory of intermolecular interactions, where additional thermal energy helped to overcome the cohesive forces between acid and oil molecules, facilitating phase separation.^38^ It should be noted that temperature variations were carefully monitored and controlled to ensure consistent aging times across samples, thereby minimizing potential confounding effects related to thermal equilibration.

Finally, the oil/acid ratio was confirmed as the most significant parameter affecting the emulsion stability. This effect was closely linked to the role of asphaltenes, which acted as natural emulsifiers by stabilizing water-in-oil (W/O) emulsions, especially at higher oil contents. As the oil/acid ratio decreased, the system shifted closer to or beyond the emulsion inversion point, where the dominant emulsion type transitioned from W/O to oil-in-water (O/W). This inversion was critically influenced by the concentration and nature of stabilizers, such as asphaltenes. Compared to previous studies on emulsified acid systems using external stabilizers,^38–40^ our findings highlighted that even without additives, operational conditions alone could govern emulsion stability—an advantage in terms of cost and simplicity. However, a potential drawback was that naturally occurring stabilizers, such as asphaltenes, might exhibit variability between crude oils, which could limit the generalization of these trends without further validation across different crude types. Understanding the interplay between the emulsion inversion point and the type of stabilizing agents is essential for predicting and controlling the emulsion behavior during acidizing treatments.

The experimental data obtained were analyzed using STATISTICA 7.0 software, which generated an empirical mathematical model based on data regression to predict the Emulsion Stability Index (ESI) at any point between the minimum and maximum limits of the three investigated variables. The resulting empirical model had a coefficient of determination (*R* ^2^) of 99.05%, indicating high accuracy in predicting values within the area defined by the experimental design. Table 3 details the variables and their interactions, presenting the effect values, coefficients, standard deviations, and *p*-values for each analyzed factor.

**Table 3:** Estimated Effects of Key Factors and Their Interactions on the Emulsion Stability Index (ESI)

parameter	effect	coefficient	standard deviation of coefficient	*p*-value
mean	42.01	42.01	0.81	0.00
FeCl_3_ (ppm)	–4.20	–2.10	0.95	0.15
*T* (°C)	–3.90	–1.95	0.95	0.17
*R*_oil/hcl_	33.90	16.95	0.95	0.00
[FeCl_3_]x[*T*]	–1.20	–0.60	0.95	0.59
[FeCl_3_]x[*R*_oil/hcl_]	–1.80	0.90	0.95	0.44
[*T*]x[*R*_oil/hcl_]	0.90	0.45	0.95	0.68
[FeCl_3_]x[*T*]x[*R*_oil/hcl_]	1.20	0.60	0.95	0.59

Equation 2 was formulated as an empirical expression to predict the Emulsion Stability Index (ESI) at points within the range of maximum and minimum values investigated. The equation was developed based on the information provided in Table 3, calculating the ESI as the sum of a constant and the variables weighted by their respective coefficients.

$$\mathrm{ESI}\left(\%\right)=42.018-2.1\left[{\mathrm{Fe}}_{3}\right]-1.95\left[T\right]+16.95\left[R_{\mathrm{oil}/\mathrm{hcl}}\right]-0.6\left[{\mathrm{FeCl}}_{3}\right]\left[T\right]-0.9\left[{\mathrm{FeCl}}_{3}\right]\left[R_{\mathrm{oil}/\mathrm{hcl}}\right]+0.45\left[T\right]\left[R_{\mathrm{oil}/\mathrm{hcl}}\right]+0.6\left[{\mathrm{FeCl}}_{3}\right]\left[T\right]\left[R_{\mathrm{oil}/\mathrm{hcl}}\right]$$ 2

In this equation, the constant and the oil-to-acid ratio (*R*_oil_/HCl) were identified as the most significant variables for the mathematical model, playing a crucial role in determining the stability of the emulsion. The constant of 42.018 represents the baseline ESI, while the oil-to-acid ratio had a significant positive coefficient (16.95), indicating that a higher proportion of oil relative to the acid tended to increase the emulsion stability. This phenomenon was attributed to the greater presence of asphaltenes and other compounds in the oil that facilitate emulsification when more oil is available to interact with the acid.^41,42^

Conversely, the concentration of ferric chloride (FeCl_3_) had a negative coefficient (−2.1), indicating that an increase in the FeCl_3_ concentration decreased emulsion stability. Temperature also had a negative coefficient (−1.95), suggesting that an increase in temperature diminished emulsion stability. The increase in thermal energy caused enhanced molecular mobility of the acid and oil molecules, reducing intermolecular interactions and facilitating phase separation.^43^

Moreover, the interaction term between FeCl_3_ and temperature (−0.6) indicated that the combined effect of these factors further reduced the emulsion stability. This behavior was attributed to the increased solubility and ionic mobility of Fe^3^^+^ ions at elevated temperatures, which enhanced their interaction with polar functional groups in asphaltenes and resins. These interactions promoted the aggregation and precipitation of the asphaltenic material, leading to the disruption of interfacial films and destabilization of the emulsion. The interaction term between FeCl_3_ and the oil-to-acid ratio (−0.9) suggested that while the presence of FeCl_3_ decreased emulsion stability, a higher proportion of oil mitigated this effect. This occurred because the greater availability of hydrophobic domains favored the stabilization of reverse micelles, counteracting the destabilizing effect of ionic species in the aqueous phase. The interaction term between the temperature and the oil-to-acid ratio (0.45) showed that a higher proportion of oil relative to the acid tended to mitigate the negative influence of the temperature on the emulsion stability. A greater amount of oil helped stabilize the emulsion, even at high temperatures. Finally, the three-way interaction term among FeCl_3_, temperature, and the oil-to-acid ratio (0.6) indicated that the combined effect of these three factors resulted in increased emulsion stability, reflecting the complex interaction among them.^7^

The validity of the model was confirmed through analysis of variance (ANOVA) and the *F*-test, with the results presented in Table 4. The calculated F1 value (44.550) exceeded the tabulated F7,3 value (5.011), while the calculated F2 value (1.137) was lower than the tabulated F1,2 value (18.51). These results confirmed that the model was predictive and significant, allowing for the accurate prediction of ESI within the range of investigated factors without the need for additional experiments.

**Table 4:** ANOVA for the Mathematical Model Predicting the Emulsion Stability Index (ESI)

source	sum of squares (S)	degrees of freedom (df)	mean square (MS)	*F*-value
model	2377.98	7	339.711	F_1_
residual	22.876	3	7.625	44.550
lack of fit	8.296	1	8.296	F_2_
pure error	14.580	2	7.29	1.137
total SS	2400.85	10		
*F* table (95% confidence interval)				
	Fcal/*F* table		model	
*F* (7.3) = 8.89	5.011		significant	
*F* (1.2) = 18.51	0.061		predictive	

The following images illustrate the interaction between the variables of ferric chloride and temperature (Figure 3A), oil-to-acid ratio and ferric chloride (Figure 3B), and temperature and oil-to-acid ratio (Figure 3C). It is evident that an increase in the temperature and ferric chloride concentration, as well as a decrease in the oil-to-acid ratio, results in a reduction in the Emulsion Stability Index (ESI). This phenomenon indicates a lower amount of acid emulsified in the oil, as previously explained.

**Figure 3:**
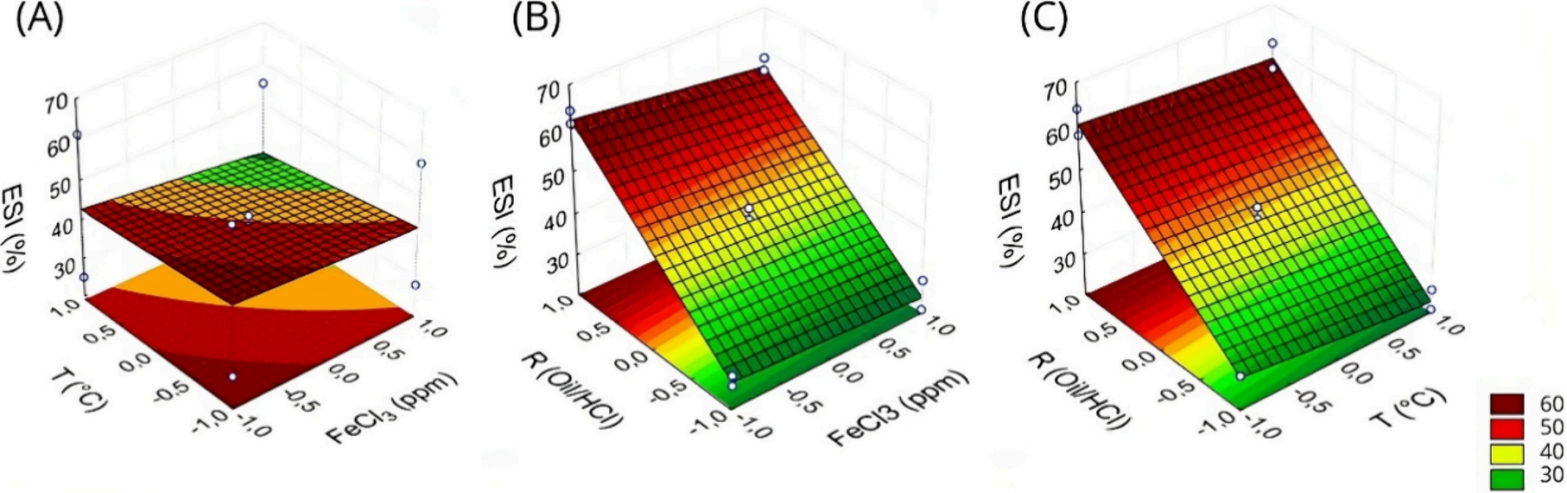
Response surfaces for Emulsion Stability Index (% ESI) for (A) FeCl_3_ vs temperature (*T*); (B) oil-to-HCl ratio vs FeCl_3_; and (C) temperature vs oil-to-HCl ratio.

### 3.2 Optimal Point Study

Figure 4 illustrates the visual results of experiments, highlighting in subfigure (B) the outcome of experiment 4, which yielded the lowest observed Emulsion Stability Index (ESI). This result indicated that experiment 4 achieved the greatest separation between the oil and acid phases, establishing it as the optimal point for analysis. Therefore, this point was adopted as a reference for investigating the effects of corrosion inhibitors, demulsifiers, and salinity.

**Figure 4:**
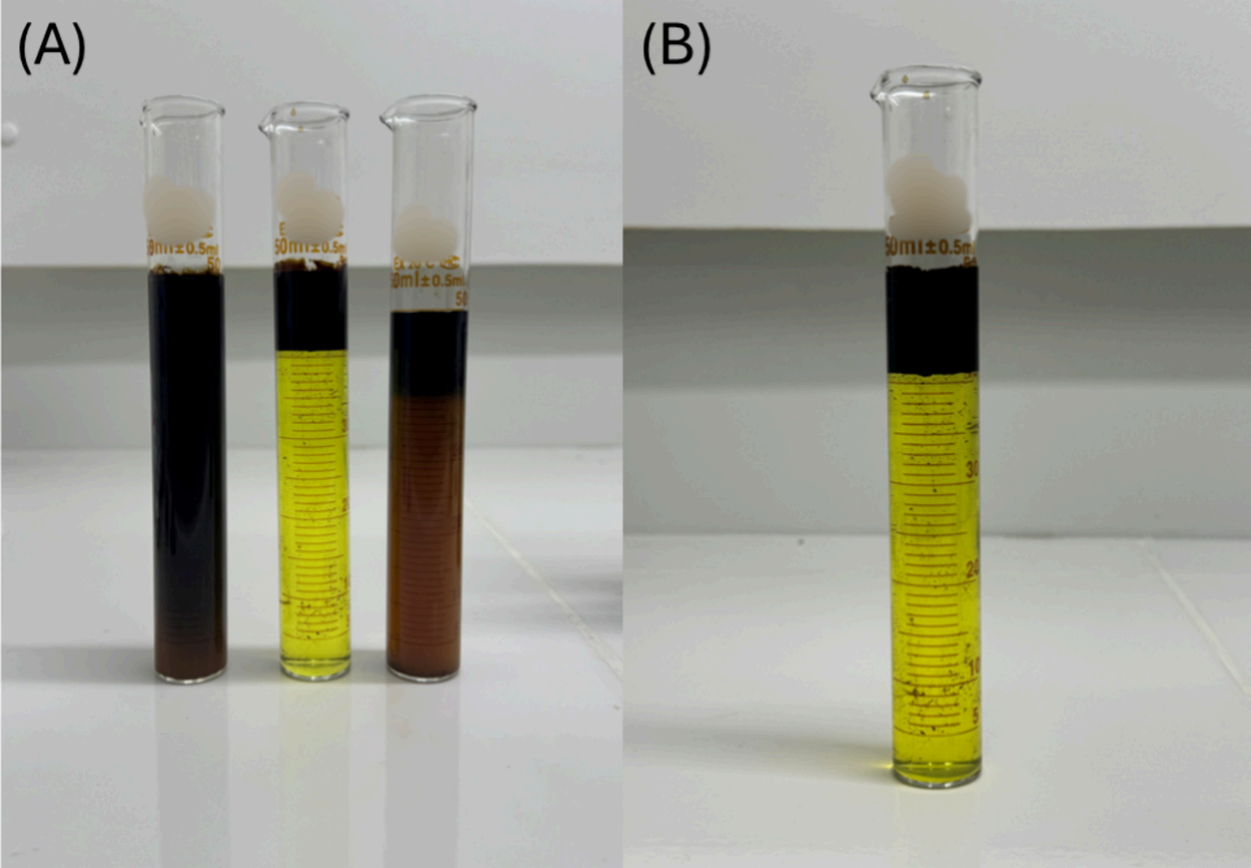
Photographic record of the emulsification tests. (A) Side-by-side images of experiments conducted under different combinations of FeCl_3_ concentration, temperature, and oil-to-acid ratio (*R*_oil_/HCl). (B) Highlighted image of the optimal conditions (experiment 4), obtained at 3000 ppm FeCl_3_, 80 °C, and *R*_oil_/HCl = 0.2.

#### 3.2.1 Demulsifier Concentration

Table 5 presents the results of the Emulsion Stability Index (ESI) concerning various concentrations of the demulsifier that were analyzed. The introduction of the demulsifier into the system at the optimal point resulted in a significant reduction in emulsion formation, with the ESI decreasing to values between 2.38 and 4.76 vol %, compared to the 20.8 vol % ESI observed at the optimal point without the use of the demulsifier.

**Table 5:** Concentrations of Emulsion Preventer (vol %) and Experimental Results for ESI (%)

emulsion preventer (vol %)	ESI (%)
1	4.76 ± 0.00
2	4.76 ± 0.00
3	4.76 ± 0.00
4	2.38 ± 0.00
5	2.38 ± 0.00

The observed decrease in emulsion stability with the use of the demulsifier was attributed to the highly polar nature of this surfactant. Demulsifiers function at the interface between the aqueous and oil phases to incorporate the smaller phase into the larger one. However, when they fail to achieve this incorporation, micellar rupture occurs. Specifically, the demulsifier used exhibited a high hydrophilic–lipophilic balance (HLB) value, characterizing the surfactant as having a strong affinity for water and a low affinity for oil.^44^ Consequently, a significant amount of oil remained outside the micellar structure, leading to the emulsion breakage. This behavior aligned with the findings of Du et al.,^45^ who highlighted that surfactants with high HLB values tend to stabilize aqueous phases and promote emulsion separation by minimizing interaction with the oil phase.

Despite its polar nature, the demulsifier contained an apolar component that allowed for the incorporation of a small amount of oil. This characteristic could lead to darkening of the lower phase, as small amounts of oil were transferred to the aqueous phase. The explanation for this phenomenon lay in the presence of an apolar portion in the surfactant, which could superficially interact with the oil and thereby contribute to its migration into the acidic phase, as discussed by Roberts et al.^46^

Figure 5 illustrates the test with a 5 vol % demulsifier, highlighting the lowest Emulsion Stability Index. A similar behavior was observed in the test with a 4 vol % concentration, indicating that the stability of de-emulsification began at this minimum concentration.

**Figure 5:**
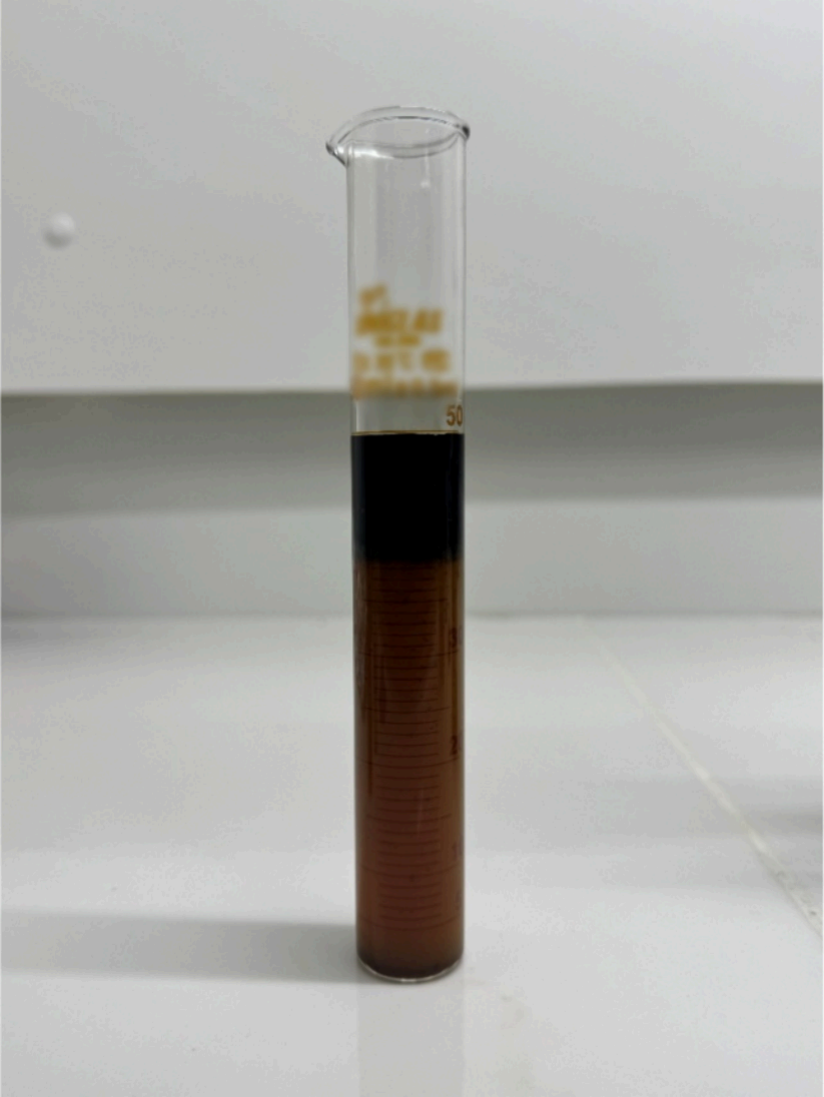
Optimal system following the addition of 5% (v/v) demulsifier.

#### 3.2.2 Corrosion Inhibitor Concentration

Table 6 presents the results of the Emulsion Stability Index (ESI) as a function of varying corrosion inhibitor concentrations. Similar to the demulsifier, the addition of the corrosion inhibitor resulted in a significant reduction in the stability of the formed emulsion. Compared to the optimal point, which had exhibited an ESI of 20.8 vol %, the use of the corrosion inhibitor decreased the ESI to a range of 7 to 11 vol %, indicating a reduction in the amount of emulsion formed.

**Table 6:** Concentrations of Corrosion Inhibitor (vol %) and Experimental Results for ESI (%)

corrosion inhibitor (vol %)	ESI (%)
1	11.9 ± 0.00
2	9.52 ± 0.00
3	9.52 ± 0.00
4	7.14 ± 0.00
5	7.14 ± 0.00

The surfactants present in the corrosion inhibitor possessed a high HLB (hydrophilic–lipophilic balance) value, although not as high as that of the surfactant used in the demulsifier. This difference in polarity affected the surfactants’ ability to interact with both the oil and aqueous phases. For the corrosion inhibitor surfactants, a high HLB indicated a greater affinity for water while still maintaining significant interaction with the oil. Consequently, these surfactants could attract and incorporate a considerable amount of oil into the micellar structure. This led to reduced efficiency in breaking the emulsion, as the presence of oil within the micelle diminished the effectiveness of phase separation.^47,48^

Figure 6 illustrates the experiment with a 5 vol % corrosion inhibitor, demonstrating that this system exhibited the lowest ESI, reflecting reduced emulsion formation. The darker appearance of the acidic phase observed in the experiment was attributed to the transport of a small amount of oil droplets into the acidic medium. This transport was facilitated by the surfactants present in the corrosion inhibitor. On the other hand, a greater amount of oil was observed separated in the upper phase compared to the optimal point without the corrosion inhibitor, which supported the increase in emulsion breakdown.

**Figure 6:**
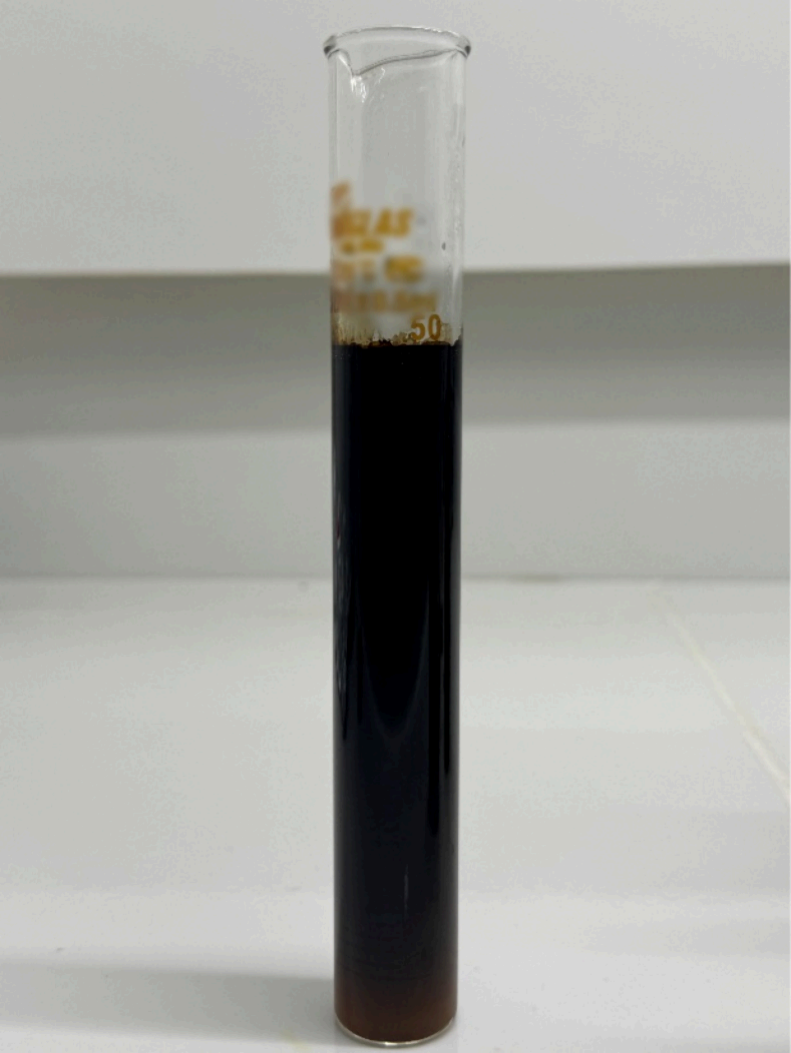
Optimal system after the addition of 5% (v/v) corrosion inhibitor.

#### 3.2.3 Effect of Salinity

Table 7 presents the results of the Emulsion Stability Index (ESI) as a function of varying salt molarities, ranging from 0.05 to 1 M, for sodium chloride (NaCl), magnesium chloride (MgCl_2_), calcium chloride (CaCl_2_), and potassium chloride (KCl). ESI was a crucial metric for assessing the stability of emulsions, reflecting the ability of the emulsion to maintain its physicochemical properties over time.

**Table 7:** Results of Different ESI values (%) as a Function of Varying Molar Concentrations of Different Salts (0.05, 0.1, 0.5, and 1 M)

	ESI (%)			
salt molarities (mol/L)	NaCl	CaCl_2_	KCl	MgCl_2_
0.05	23.8 ± 0.00	16.67 ± 0.00	21.42 ± 0.00	21.43 ± 0.00
0.1	16.67 ± 0.00	14.28 ± 0.00	19.05 ± 0.00	16.67 ± 0.00
0.5	16.67 ± 0.00	14.28 ± 0.00	19.05 ± 0.00	14.28 ± 0.00
1	16.67 ± 0.00	9.52 ± 0.00	16.67 ± 0.00	14.28 ± 0.00

For a molarity of 0.05 M sodium chloride, the observed ESI was 23.8 vol %, representing an increase of approximately 3 vol % compared to the ESI of the optimal point used as a reference. Although a significant decrease in ESI was observed from 0.05 to 0.1 M NaCl, the values then remained constant up to 1 M, indicating a plateau effect. This plateau suggested that once a critical ionic strength was reached (∼0.1 M NaCl), additional ions no longer enhanced the destabilization of the emulsion. At this point, the electrostatic double layer surrounding the droplets had already been sufficiently compressed, limiting further reductions in the electrostatic repulsion. In other words, beyond this threshold, additional Na^+^ and Cl^–^ ions contributed little to further destabilization because the system approached an electrostatic equilibrium at the oil–water interface.

This behavior was also consistent with ion-specific hydration effects. At low to moderate concentrations, Na^+^ and Cl^–^ ions preferentially interacted with water molecules, thereby reducing the hydration shell around the asphaltene heteroatoms. This diminished the surfactant-like behavior of asphaltenes and promoted destabilization. However, once the hydration preference of water molecules was saturated, further increases in NaCl concentration had minimal additional impact, explaining the plateau.

This phenomenon could be explained by the dynamics of chemical interactions among the emulsion components. Sodium chloride dissociated into Na^+^ and Cl^–^ ions in the solution, which could affect the emulsion stability by altering the ionic strength of the medium. An increase in NaCl concentration elevated the ionic strength of the solution, which screened the existing electric double layer around the emulsion droplets. This screening effect reduced electrostatic repulsion between the droplets, thereby promoting coalescence.^49,50^

Additionally, hydration effects occurred, where water molecules hydrated H^+^, Na^+^, and Cl^–^ ions. Water molecules naturally interacted with the free electrons present in the heteroatoms that compose the asphaltene fractions of the oil. When saline ions were introduced into the medium, water preferentially hydrated these ions due to the more favorable nature of this interaction. The decrease in interaction between water and asphaltenes led to a reduction in the surfactant activity of these molecules, which in turn resulted in diminished emulsion stability.^51^

In the case of KCl, a similar plateau behavior was observed between 0.1 and 0.5 M, followed by a decrease in ESI at 1 M. This indicated that, as with NaCl, electrostatic compression and hydration competition reached equilibrium at intermediate concentrations, preventing further destabilization. However, at higher concentrations (1 M KCl), additional ion-specific effects, such as the larger ionic radius and lower hydration energy of K^+^ compared to Na^+^, may have facilitated ion accumulation at the interface. This could further disrupt interfacial film stability, explaining the observed secondary decrease observed.

The same explanation was applied to the other salts studied. Magnesium chloride (MgCl_2_), calcium chloride (CaCl_2_), and potassium chloride (KCl) exhibited similar behaviors, influencing emulsion stability according to the ionic strength they introduced into the solution. The presence of these salts altered the electrostatic properties of the emulsion, potentially leading to decreased emulsion stability with increasing concentration of each of these salts, as described by Qi et al.^52^ Nevertheless, the experimental data confirmed that after an initial destabilization, ESI values tended to plateau, suggesting the establishment of an interfacial equilibrium. Only at sufficiently high ionic concentrations (e.g., 1 M KCl) did specific ion effects overcome this balance, leading to renewed destabilization. It remained unclear whether concentrations above 1 M, such as 2 M NaCl or MgCl_2_, would further reduce the ESI or simply reinforce the plateau, and this warrants further investigation in future studies.

## 4 Conclusions

Based on the results obtained from the conducted experiments, it has been established that the oil-to-acid ratio is a critical factor in the formation of emulsions. An increased oil proportion relative to acid promotes enhanced interaction between H^+^ ions and the polar fractions of crude oil, particularly asphaltenes, leading to greater emulsion stability. This behavior is significant in the analysis and optimization of reservoir acidizing processes and directly informs operational decisions in field-scale treatments, where minimizing emulsion formation can reduce downtime, improve acid efficiency, and lower operational costs.

Additionally, it was identified that the points of minimal emulsification occur when the temperature is at its maximum, specifically 80 °C; the concentration of FeCl_3_ is at its maximum, around 3000 ppm; and the acid-to-oil ratio is at its minimum, i.e., 0.2. These parameters were shown to be the most effective in minimizing emulsion formation, which is undesirable in acidizing processes as it can compromise treatment efficiency and increase acid consumption —a critical concern for field operations aiming to optimize throughput and chemical usage.

Although temperature and FeCl_3_ concentration are not of extreme importance when considered individually, their simultaneous reduction significantly increases emulsion formation, as lower thermal energy and diminished FeCl_3_ content limit destabilization mechanisms, thereby increasing the amount of acid that emulsifies in the oil. Although the proposed model demonstrates high predictive power within the studied parameter space, its application in industrial scenarios requires consideration of reservoir heterogeneity and scale-up factors; future studies should therefore investigate its applicability across a wider range of HCl concentrations and for crude oils with varying physicochemical characteristics to ensure robust field deployment.

In light of these observations, it is recommended that future studies explore the variation and interaction of these parameters to optimize the acidizing process. In particular, further investigation is warranted into the influence of asphaltene content and emulsification mechanisms, including the identification of the type of emulsion formed (oil-in-acid or acid-in-oil) as well as its inversion behavior. Furthermore, it is essential to investigate other additives and operational conditions that might contribute to reduced emulsification, enabling more reliable and cost-effective acidizing treatments under realistic field conditions. Comparative studies with recent developments in the literature, such as emulsified acid systems using nanomaterials or organoclays, may also yield valuable insights into the design of more environmentally responsible and operationally effective acidizing fluids. Such an integration of laboratory findings with industrial practice can guide the development of new formulations and techniques that enhance both the performance and sustainability of carbonate reservoir treatments. Nevertheless, this study is limited by the absence of advanced crude oil characterization techniques (e.g., SARA analysis) and field-scale validation experiments. These constraints do not diminish the validity of the observed trends but should be considered when extrapolating the findings to heterogeneous reservoir conditions. Future work addressing these aspects will further strengthen the robustness and applicability of the proposed recommendations.

From a practical standpoint, the findings presented here provide actionable guidance for field engineers and operators by identifying operational parameters—such as optimized oil-to-acid ratios, temperature control, and ferric ion concentration management—that can be directly adjusted during matrix acidizing to minimize emulsion formation without relying on costly or complex chemical additives. These insights support the more efficient use of acid, reduce the risk of formation damage, and enhance overall treatment performance. Looking forward, integrating these operational strategies with emerging technologies, such as nanomaterial-based stabilizers or advanced demulsification techniques, offers promising avenues for further improving both the economic and environmental sustainability of carbonate reservoir stimulation.
